# Coping with COVID-19 Intensive Care Unit admission

**DOI:** 10.1590/0034-7167-2025-0382

**Published:** 2026-07-24

**Authors:** Mariane Carolina de Almeida, Maria Itayra Padilha, Maria Lígia dos Reis Bellaguarda, Stéfany Nayara Petry Dal Vesco, Jaime Alonso Caravaca-Morera

**Affiliations:** I Universidade Federal de Santa Catarina. Florianópolis, Santa Catarina, Brazil; II Universidade da Costa Rica. San José Province, San Pedro, Costa Rica

**Keywords:** Coping Skills, Coronavirus Infections, Post-Acute COVID-19 Syndrome, Nursing, Intensive Care Units., Habilidades de Afrontamiento, COVID-19, Síndrome Post Agudo de COVID-19, Enfermería, Unidades de Cuidados Intensivos.

## Abstract

**Objectives:**

to analyze, in light of Folkman and Lazarus’s Coping Theory, the reflections on life expressed in experience reports of patients hospitalized in an Intensive Care Unit for COVID-19.

**Methods:**

qualitative research, using Thematic Oral History. Ten patients who were hospitalized in a COVID-19 Intensive Care Unit of a university hospital in southern Brazil between 2020 and 2021 were interviewed. Analysis was based on Minayo’s theoretical model.

**Results:**

six men and four women participated, with an average age of 52 years. Three categories emerged, “Post-COVID-19 sequels”; “Quality of care and infrastructure”; and “Value of life and change of perspective”.

**Final Considerations:**

during hospitalization, the importance of healthcare professionals, especially nursing professionals, was highlighted. A change in future perspective was identified, with a greater appreciation for family closeness and a greater appreciation for life. After the illness, difficulties related to memory, anxiety, fatigue, and cognitive dysfunctions were identified.

## RESUMEN

**Objetivos:**

analizar, a la luz de la Teoría de Afrontamiento de Folkman y Lazarus, las reflexiones vitales expresadas en los relatos de experiencias de pacientes hospitalizados en la Unidad de Cuidados Intensivos por COVID-19.

**Métodos:**

investigación cualitativa, mediante Historia Oral Temática. Se entrevistaron a diez pacientes hospitalizados en la Unidad de Cuidados Intensivos por COVID-19 de hospital universitario del sur de Brasil entre 2020 y 2021. El análisis se basó en el modelo teórico de Minayo.

**Resultados:**

participaron seis hombres y cuatro mujeres, con una edad promedio de 52 años. Se identificaron tres categorías: “Secuelas pos-COVID-19”; “Calidad de la atención e infraestructura”; y “Valor de la vida y cambio de perspectiva”.

**Consideraciones Finales:**

durante la hospitalización, se destacó la importancia de los profesionales sanitarios, especialmente de enfermería. Se identificó un cambio en la perspectiva de futuro, con una mayor apreciación de la cercanía familiar y una mayor valoración de la vida. Tras la enfermedad, se identificaron dificultades relacionadas con la memoria, ansiedad, fatiga y disfunciones cognitivas.

## INTRODUCTION

During the COVID-19 (coronavirus) pandemic, a disease caused by the SARS-CoV-2 virus^(1,2)^, the world population suffered from the fear of contracting the disease and dying, from the consequences of self-isolation, from the economic impact, etc. In short, the challenge was immense. In addition to the often-nonspecific signs and symptoms^(3)^, individuals who contracted COVID-19 began to present physical and emotional sequels, known as post-COVID-19 syndrome^(4)^. Among the most frequently cited sequels in the world literature are persistent anosmia, dyspnea during activities of daily living, short-term memory loss, and mental health problems such as depression, anxiety, and post-traumatic stress disorder^(4,5)^.

Faced with these challenges, nursing has been present at all levels of healthcare, providing care to patients throughout the course of their illness^(6)^. Nurses were seen working on the front lines in Basic Health Units, COVID-19 Screening Centers, Emergency Care Units, field hospitals, other hospitals, and other health settings^(7-9)^. In hospitals, nursing staff had to adapt to the new COVID-19 sector, which often provided care in both internal medicine and Intensive Care Unit (ICU)^(10,11)^.

Nurses worked in harsh conditions in ICUs, facing overload and increased working hours, lack of supplies and human resources as well as the fear of contracting the disease and being stigmatized if they became infected^(12)^. Even with all these difficulties, it was possible to work with a humanized approach and maintain quality of care. Although stress related to the pandemic often occupied people’s minds, it is clear that society learned a great deal during this period. This is due to the recognition of healthcare professionals’ work, the increase in acts of solidarity, and the heightened appreciation for the importance of family. In short, it seems that life has been given more value^(4,13-16)^.

In this context, considering this entire process of stress, (un)certainty, resilience, and learning post-COVID-19, there is Folkman and Lazarus’s Coping Theory^(17)^, which refers to the strategies that individuals employ in stressful situations. Throughout the COVID-19 pandemic, there was stress related to self-isolation, fear of infection, fear of death, fear of hospitalization, and fear of needing intensive care. There were various stressors during the pandemic. Faced with these situations, individuals create coping strategies. Some of these strategies may focus on emotion, and others on the problem^(18)^.

Although the physical and emotional consequences of COVID-19 on individuals and the professional role of nursing during the pandemic have been widely documented, studies exploring, from the perspective of Folkman and Lazarus’s Coping Theory, how patients admitted to ICUs made sense of their experiences and developed coping strategies in the face of the severity of the disease and its sequels are still scarce. This prompts the question: how do patients admitted to ICUs for COVID-19 in a university hospital in southern Brazil express, in light of Folkman and Lazarus’s Coping Theory, their coping capacities and coping strategies?

## OBJECTIVES

To analyze, in light of Folkman and Lazarus’s Coping Theory, the reflections on life expressed in experience reports of patients hospitalized in an ICU for COVID-19.

## METHODS

### Ethical aspects

The study was conducted in accordance with national and international ethical guidelines and approved by the *Universidade Federal de Santa Catarina* Research Ethics Committee, whose opinion is attached to this submission. It was developed in accordance with Resolution 466/2012 of the Brazilian National Health Council. All participants signed the Informed Consent Form.

### Study design

This study adopted a qualitative approach, with a narrative-socio-historical design, using oral sources through Thematic Oral History^(19)^. The methodological process was improved following the Consolidated criteria for Reporting Qualitative research checklist^(20)^, ensuring greater rigor and specificity.

### Study setting

It was developed in a university hospital in a municipality in the state of Santa Catarina (SC), which opened 78 years ago. It is considered a strategic hospital by the Ministry of Health, being a point of attention in the Emergency and Urgent Care Network. It plays a prominent role in the macro-regional health community of Joaçaba, SC, being a reference in the specialties of oncology and neurosurgery for more than 55 municipalities, totaling more than 600,000 inhabitants. It has a capacity of approximately 200 beds. It has a general ICU with 20 beds and a COVID-19 ICU with ten beds. This latter ICU was opened in March 2020, due to the needs of patients with COVID-19, and its activities ended in May 2022. The COVID-19 ICU had ten adult beds, separated by bed divider curtains. The nursing station was located in the center of the unit. Each bed had a hospital bed, a parametric monitor, a mechanical ventilator, infusion pumps, a support table, and a compressed air and oxygen supply system.

### Study participants

Ten individuals who were hospitalized in the COVID-19 ICU of *Hospital Universitário Santa Terezinha* in Joaçaba, SC, between 2020 and 2021 participated in the study. Patients included in the research were those who tested positive for COVID-19 and were hospitalized in the COVID-19 ICU of this hospital, were over 18 years of age, and resided in the city of Herval d’Oeste, SC. Patients hospitalized in the COVID-19 ICU of this hospital who resided in other cities and those with cognitive impairments that prevented participation in the interview were excluded from the research. Patients residing in the city of Herval d’Oeste, SC, which is part of the Joaçaba macro-regional health area, were invited to participate in the study. Access to case notifications was obtained through the epidemiological surveillance sector of the municipality of Herval d’Oeste, SC. Participants were intentionally selected.

### Data collection and organization

Data collection was carried out using a semi-structured interview guide, applied to participants in a hybrid format. Data collection was conducted by the principal researcher. Two participation formats were offered-online or in-person-at their homes or workplace. Five participants preferred to answer the interview online, and five preferred in-person. The questions addressed experiences of people hospitalized with COVID-19, especially in the ICU. They discussed personal, professional, and health life before the illness, as well as feelings and experiences during hospitalization, readjustment after discharge, and current impacts, including sequels, life changes, and family support in coping with the illness. All interviews were recorded using the Zoom^®^ application with the prior authorization of participants. The interviews took place between June 2023 and June 2024. After transcribing the data in Microsoft Word^®^, the files were sent to participants via WhatsApp^®^ so that they could validate the interview.

### Data analysis

Subsequently, data analysis was carried out as proposed by Minayo^(21)^, divided into two stages: organization and exploratory phase; and interpretation and thematic analysis. A careful and exhaustive reading of the data was performed to obtain an overview and establish initial relationships, ensuring internal coherence. Preliminary perceptions were recorded, and a critical data analysis was initiated, maintaining the consistency of information. During data exploration, coding was performed, allowing the formation of recording units. The most frequent codes with the greatest similarity in meaning contributed to categorization. This stage included grouping the registration units into subsets based on their similarity and correlation. The analysis was aligned with Coping Theory. From the analysis, three main categories emerged: “Post-COVID-19 sequels”; “Quality of care and infrastructure”; and “Value of life and change in perspective on the future”. To preserve participant anonymity, alphanumeric codes (P1, P2, P3, etc.) were used, assigned in chronological order of the interviews.

## RESULTS

The study included ten individuals, six male and four female. Participants’ ages ranged from 34 to 71 years, with an average of 52 years. Concerning race/color, six identified as white and four as mixed-race. As for marital status, seven were married, two were single, and one was widowed when contracting COVID-19. The predominant religion was Catholicism, with nine representatives, followed by Protestantism. The average length of stay in the ICU was 11.6 days, with a minimum stay of four days and a maximum of 33 days.

### Post-COVID-19 sequels

Reports on the physical and psychological difficulties faced after COVID-19 infection highlight the challenges participants experience even after initial recovery. In addition to physical after-effects, many research participants reported anxiety, depression, and post-traumatic stress disorder due to their experiences during infection and hospitalization.

*For example, just yesterday I asked someone for their name, and ten minutes later I had already forgotten it, so about forgetfulness* [...] *it’s complicated for me to remember a lot of things, but it was a very difficult moment.* [...] *my breath, if I overdo it, it becomes difficult to go up or down stairs quickly. I already feel the difference. Of course, it’s not 100%, right? So, I can’t do an exercise that demands too much of me, right? Today, I go for walks, cycle, but if I try to play ball, I take a breather because I get tired in two touches.* (P2)

*Today, I have a problem because I read ten pages, and then I go back and forth; I can no longer tell if it was a chapter or a short story; I can’t keep up anymore. And that’s one of the difficulties I have with series. I’ve lost a bit of that memory. I don’t have the ease of remembering anymore, and with reading, several times I’ve had to restart or reread the chapter. I’ve lost a bit of the old memories.* (P3)

*First of all, how can I put it? You lose physical stamina; you want to walk, and any effort makes you sick, you have difficulty breathing, at least I do, and I get forgetful. Heart problems appeared, my blood pressure never goes down, even with medication, every now and then I have to go to the emergency room because it doesn’t go down.* [...] *I feel very anxious, and it’s the same as when I was in the hospital.* (P5)

*After COVID-19, my body has never been the same. I’m always tired, and this tiredness seems like it will never go away. Even doing the simplest tasks, like getting out of bed or making lunch, leaves me exhausted. My mind isn’t working like it used to either; sometimes it freezes up, and there are days when I feel completely lost. I forget important things and take longer to do tasks that used to be easy for me. It’s as if my mind is constantly slow and heavy.* (P7)

*My time with COVID left me very tired, with no energy for anything. I felt extremely exhausted and my memory didn’t seem to work as well as before. I needed physiotherapy to regain my strength, and I had psychological support both in the hospital and at home. It was a slow and often tiring process, but I felt grateful for every small progress I made. The fatigue makes simple daily tasks difficult, and I feel like my memory isn’t what it used to be.* [...] *I also felt very anxious and sad during the recovery process, and I needed support from a psychologist. Nightmares and the fear that something bad could happen again were frequent at the beginning, but therapy and the support of my family have helped me a lot to overcome this.* (P8)

Reports show that even after recovering from COVID-19, participants face physical and psychological challenges that compromise their quality of life. Symptoms such as fatigue, breathing difficulties, memory loss, and cognitive slowness are common, as are anxiety, fear, and post-traumatic stress. These impacts affect daily life and mental health, requiring ongoing monitoring and coping strategies, such as therapy and family support.

### Quality of care and infrastructure

Patients’ perceptions of nursing services and hospital infrastructure during the COVID-19 pandemic offer important insights into the quality of care and the conditions faced during the pandemic. During hospitalization, nursing professionals used words of comfort, encouragement, and hope with patients, in addition to demonstrating humanized care, as expressed by participants during moments of changing clothes, feeding, and monitoring vital signs. Furthermore, study participants reported attitudes of constant presence, caring touches, attentive gazes, and listening postures that reinforced humanization of care.

*There was one day I woke up like this* [...] *around 3:30 in the morning, soaked in sweat; nurses were changing me because I was all wet from my oxygen saturation dropping.* [...] *but their care was very good, the food, the attention, there’s no denying that. I wasn’t intubated, but I had that mask that went over my nose and mouth, you know. Even so, nobody deserves that, I couldn’t get any air.* [...] *the healthcare staff did nothing to bridge that distance; the only thing they did was call a family member to let them know how things were going. They would ask, “Who do you want me to tell so we can pass on the message?” They would relay information about how the person was doing or not. That was the only way.* [...] *they could have treated people better. Some, and I always say “some” because you can’t generalize; there are doctors there who we still remember as good people, and others, well, it’s better not to even mention them, but the care itself was excellent.* (P2)

*She ate well; it was even motivating when someone came in with a tray. That’s how it was. In fact, the nurse’s treatment was great-I even wrote a text once for a nurse who had collaborated with us; she was the health secretary, and a nurse is someone who takes care of you, who puts you to bed and asks if you’re okay.* [...] *the doctor is fundamental, but the person who deals with you, who asks to change patients, is the nurse. Nursing is undervalued financially; primary school teachers and nurses, I think, earn little. The medical system was part of treatment, but the psychologist or a social worker made the video call. Professionals helped a lot by talking, sharing life stories, where you came from, I think that helps a lot. Perhaps this other approach is not widely known in nursing. I received a lot of support; doctors are a bit more technical, but the nursing and physiotherapy staff were more human.* (P3)

*I remember a nurse asking if I could take a shower, and I said I could. So, I went in to take a shower, but no, I couldn’t. I started having trouble breathing, I started feeling unwell, and the nurse had to go and help me finish the shower. I was on oxygen via catheter, then it was changed and I was given that mask with the bag underneath, and from there I went to the ward, to the COVID ward* [...]. *There came a point where there was only one nurse in the ICU monitoring the equipment, and then the rest would come in to help, to take a look, because outside, I even remember a phrase the nurse told me, “It looks like a war zone out there”.* [...] *in terms of the healthcare staff, well... ten out of ten.* [...] *there was also a psychologist there who talked to us. It was good; she talked, asked how I was doing, and touched on family matters; it helped to distract us a little during that time there, she always told us to think about good things, it was really nice. Actually, within the healthcare field, during the time I was there, it was first-rate care, the dedication of everyone* [...] *from the doctors, nurses, technicians, the psychologist, the physiotherapist, the staff who provided care, who brought snacks, the cleaning staff, everyone.* (P5)

*Professionals did everything to make us feel welcome, and that made all the difference. They were wonderful, always offering a kind word. There was a nurse who always said, “Everything will be alright”. That made us feel like we weren’t alone. They knew we needed support, and they gave their best. Then there was also the psychologist who made the video call; it was very good; I felt closer to my Family.* (P6)

*Inside, they were like family to me: the nurses, the doctors. They always said that everything would be alright. They knew how difficult it was to feel alone in that situation, so they did everything they could to comfort us and give us hope. Even in the saddest moments, they tried to show that there was still a chance. Their care and attention were very important for me to get through all of this.* [...] *look, in the ICU, I don’t remember exactly, but later, when I returned to the clinic, they always did everything they could to make me feel better. They were truly angels who were there to take care of us. They were real angels, always very attentive and dedicated. Even with so many sick people, they did what they could to comfort me.* (P7)

*They were a great support during my hospitalization and this period away from my family. Many of them tried to encourage me, saying that I was getting better, that I was progressing, and that gave me hope. Some nurses talked to me about everyday things, as if I wasn’t even in the hospital. I felt that God was present through these professionals who were so dedicated to caring for me and so many others. The relationship with healthcare professionals was extremely important and brought comfort amidst it all. Even though I was unconscious for a good part of the time I spent in the ICU, when I started to wake up, I noticed the care and dedication of each nurse, each doctor. They were always present; it was constant care.* (P8)

Concerning hospital infrastructure, patients shared their impressions of the environment in which they received treatment during the pandemic. The descriptions included both positive and negative aspects, highlighting the importance of an adequate and well-equipped space for healthcare.

*That’s when the battery of tests began. In that room, it was the third bed, it had no structure at all, you know? That bed in the storage room, it didn’t even have mobility. If I said I had leg pain, they would put a pillow underneath, or a rolled-up blanket. It was kind of adapted, I received visits from the doctors like that, until, the next day, I spent the whole night of the 3^rd^, and on the night of the 4^th^, I was transferred to Chapecó.* [...] *it was the third bed in a room; it was adapted from the hospital; there were other people who arrived later; there were only four ventilators, so I used oxygen via catheter. The place, there was nothing to do, it was a storage room, but not in a pejorative way, there was no room for the ventilator, no room for oxygen; some people were manually ventilating to help. Honestly, having worked in public health, I tell you: I feel there was a certain neglect in not having post-COVID programs; there wasn’t an official post-COVID program; I had hormonal, neural, and circulatory problems; I had to undergo a battery of tests. Fortunately, I have health insurance; I started looking for specialists-neurologist, endocrinologist, nephrologist, etc.; I had quite a few complications. I didn’t have these problems before, so it was post-COVID.* (P3)

*So, when I went to the hospital, I stayed in that white room until they could get me a bed. They said I might have to go to Campos Novos, but I didn’t want to go far, but luckily, “what a shame”, luckily for me, they said a man died, so there was going to be a bed available, so I stayed there, I think in the clinic for about two days, and then I didn’t see anything else.* (P7)

*Then they wanted to take me to Capinzal, and I said, “No, leave me here”. Then they did an X-ray, and they wanted to take me to Campos Novos because the hospital there didn’t have an ICU bed. I said, “Leave me here”, and I was already arguing with the men there. Then after the X-ray came back, they said, “No, we’re going to admit you here”. They said I was in bad shape; they left me downstairs on the stretcher with oxygen; they wanted to transfer me, and I don’t know if it was Monday or Tuesday, they wanted to use that mask that pushes oxygen so I could breathe better, and then they wanted to take me to Balneário Camboriú with the mobile ICU. Then I don’t know, my sister-in-law from Campos Novos knew the doctor. They tried to get a bed in the ICU there, it didn’t work out, we spoke to the doctor, who was also an angel in my life, who saw that someone had died, and then I managed to stay there.* (P10)

Reports highlight the recognition of the nursing staff’s care and the structural challenges faced. There is appreciation for the welcoming, listening, and emotional support received. Limitations related to overcrowding, improvised spaces, and difficulties in accessing ICU beds are also evident, reflecting the impacts of the health crisis on quality of care.

### Value of life and change in perspective on the future

Many participants reported changes in their outlook on life after recovering from COVID-19, and offered profound insights into the transformative impact the disease can have on individuals. These reports highlighted the increased value placed on aspects of life that were previously undervalued, such as interpersonal relationships, health, and the importance of simple everyday moments.

*Look, from a psychological standpoint, something I’ve said before and will say again: you have to enjoy life today because you don’t know what tomorrow will bring. Eat and drink because that’s what you’ll take with you. Enjoy it, because after seeing people intubated next to me, dying in my room, what I’ve learned from experience today is that you have to enjoy life. Tomorrow, you don’t know if you’ll get up, or if you’ll have a heart attack, or if a car will hit you, you understand? So, I always say, “You have to enjoy it”, because as I always say, “If you have your health, you’ll be fine”. So, that’s the lesson I’m sharing: you have to enjoy it.* (P2)

*To value life and, of course, to enjoy it more, but we are in a position here where I’m sure that, I have another six or seven months in office, and every day I think, “My biggest challenge is to be motivated every day to finish the term working the way we are, doing better”.* [...] *it’s noticeable to me, you know, to really value life, because I was, in my view, on the other side already, but I had a second chance and some things that we could be doing, doing wrong.* (P4)

*We start thinking, we value things more, things like food. You’re there, sometimes I was there in the hospital, you wanted to eat, but you couldn’t because of your condition. Everything, everything changes: your way of thinking about things. There are things that aren’t worth wasting time on, many things change a person’s opinion, making them value life more. One thing I paid a lot of attention to was, like this: the simple fact of being able to. In my case, I was in the ICU, and after I came back feeling a little better, I wanted to go to the bathroom, but I couldn’t, and you see how weak and fragile human beings are. And another thing, a person who is independent does everything, and then, out of nowhere, needing other people to help you with everything, absolutely everything, to take a shower, for everything, for physiological needs, everything changes, it’s a huge learning experience. The experience is bad, but the learning is very valuable. Look, the only thing is for people to value the people who are by their side, to value their family more and more, to enjoy their time and be happy, not to waste time on petty things, things that don’t lead them anywhere.* (P5)

*I’m grateful to be alive, to have my husband and family with me. I did a lot of physical therapy; I recovered slowly. My pets were always close by, the girls called every day* [...] *that’s what kept me strong. You learn to value every moment. A lot has changed. I think that, after going through this, you see life differently. I learned to value the little things more: health, family* [...] *now I take better care of myself, I listen to my body more. Life is so fragile; we can’t waste time on small things.* (P6)

*I think that, after going through all of that, you see life in a very different way. I’ve learned to value my health and my family much more. Before, I don’t think I gave those things so much importance, but now it’s different. We have to value what really matters: the things that make a difference and that bring joy and satisfaction.* (P7)

*I try to be closer to my family, cherishing every moment with them, and I try to enjoy life with more gratitude and presence. Many things have changed in my life. First, I realized the importance of taking better care of my health. I am more aware of the need for a healthy diet and physical exercise. I also began to value the simple moments with my family and friends more. The experience in the ICU made me reflect on the fragility of life and the importance of living in the present, with gratitude and purpose. The illness taught me the importance of never losing hope, of valuing the small victories of everyday life, and of always maintaining faith in God, even in the saddest moments.* (P8)

*My life is better than before. COVID and that time I spent in the hospital makes you think about many things, about life, about the simple things I could do but was there, disabled, it makes you think a lot about family, wow, everything is so much better.* (P10)

Reports indicate that the experience of being in the COVID-19 ICU sparked profound reflections on the fragility of life, leading participants to redefine their priorities. Valuing health, family, and simple moments became essential. The experience of depending on intensive care and facing the risk of death reinforced feelings of gratitude, motivating changes in lifestyle, with more presence, affection, and purpose.

## DISCUSSION

Based on the findings, it was possible to identify persistent sequels in patients after COVID-19. Some of these are physical, such as dyspnea, fatigue, and cardiovascular problems; others are cognitive, such as short-term memory loss; and others are emotional, such as anxiety and depression. Understanding these experiences allowed for the analysis of reflections on life by these patients who were hospitalized in the COVID-19 ICU from the perspective of Folkman and Lazarus’s Coping Theory.

The long-term effects of COVID-19 are documented in several studies, with the most frequent being fatigue, shortness of breath, palpitations, insomnia, chest pain, muscle weakness, hair loss, joint pain, abdominal pain, nausea or vomiting, muscle pain, decreased appetite, headache, olfactory disturbance, taste disturbance, and dizziness^(14-22)^. In general, these sequels are heterogeneous, meaning they affect various biological systems, although shortness of breath is one of the most frequently cited symptoms in the literature^(23,24)^. Authors have highlighted post-COVID sequels in patients who underwent critical care^(24)^, which revolved around physical, cognitive, and psychosocial function, such as weakness, prolonged respiratory symptoms, cognitive changes, post-traumatic stress disorder, anxiety, and depression. Furthermore, post-COVID sequels have proven to be a complex challenge, affecting multiple body systems, and resulting in respiratory, cardiovascular, neurological, psychological, and musculoskeletal complications^(25)^. As observed in the present study, patients reported many of the post-COVID symptoms described in literature; however, post-traumatic stress was not diagnosed or reported by participants.

Research that studied nurses who were infected with COVID-19^(26)^ observed reports of anxiety during the COVID-19 recovery period, a finding that supports the present study, since participants reported a need for psychological support and the use of medication for mental health treatment. These data highlight the importance of follow-up programs for post-COVID patients, with the aim of supporting the rehabilitation of these sequels, bringing better quality of life to these individuals.

The stress experienced by study participants denotes encouragement to search for coping strategies. Fear, anxiety, and insecurity caused stress in participants, leading to the development of unconscious mechanisms to deal with the illness. These mechanisms are considered emotional coping^(18)^, in which family and religious support were sought to cope with this process.

Studies^(27,28)^ indicated that healthcare professionals also used support and communication with family, friends, and co-workers as their main coping mechanisms. Religious coping mechanisms, such as prayer, were reported as an important coping mechanism. This study emphasizes how much nursing was praised in terms of technical competence and psychological support. Participants seem to value nursing services, emphasizing the importance of these professionals and corroborating the data from a study^(29)^ in which patients recognized nurses’ competence for their recovery from COVID-19.

Recognition directed towards the nursing staff demonstrates the importance of this professional category in the coping process. Through the bond created with patients, professionals instill confidence, becoming a point of security for these patients, and this, unconsciously, helps in the process of coping with the illness and hospitalization in the ICU^(30)^. The focus is on regulating emotions in accordance with the stress experienced and the relationships established between professionals and family members of patients, as well as among patients themselves, through mutual support, active listening, and empathy. Furthermore, religious and spiritual foundations provided support for coping, which, in contrast to the emotion-focused coping described by Folkman and Lazarus^(18)^, brings regulation to the reflective state against stress^(31)^. It should be considered that professionals were also coping during the pandemic, perhaps due to stressors different from those of patients, but which complemented the situations experienced by users^(32)^.

Some study participants also mentioned problems related to hospital infrastructure, which was generally poor. These problems cannot be addressed, as distress is also associated with poor working conditions^(30)^. The environment was described as adapted, like a “warehouse”. Patients’ anxiety regarding the uncertainty of securing a hospital bed was also evident. The fear of being far from their hometown and having to be referred to a hospital even further from their family was highlighted by the participants. Research participants^(29)^ reported fear of hospital overcrowding, medication shortages, and a lack of healthcare professionals, similarly to the individuals in the present research.

Despite the limitations presented by hospital settings, the humanization and care provided by professionals were essential for patients’ physical and emotional support. It can be inferred that patients used the support of professionals as a form of emotional coping^(18)^. Using words of support and encouragement, individuals in the study found in healthcare professionals a source of comfort in moments of stress.

Changes in perspectives on life, family, and the future were also observed. Some participants reported a greater appreciation for family time and valued the freedom to choose what to eat. In short, it became clear that patients value the simple moments in life more. These mechanisms may be involved with exposure to intense stress, given that studies^(4-13)^ portray changes in perspectives after stressful situations, such as COVID infection or even ICU admission.

Another study^(29)^ describes that participants expressed that they should not allow life stress to harm their health, and also stated that financial issues, for example, were no longer as important to them as before the illness. The hospital experience leads to a “re-signification” of the body, with reports similar to the present study of loss of resistance and changes in physical health^(16)^.

This re-signification of life can be considered a problem-focused coping strategy^(17)^, given that, after a stressful situation, patients decide to take better care of their health, reassessing the quality of their diet, the quality of their social interactions, and the importance of physical activity. These are simple solutions to the problem faced.

These findings provide support for offering education to healthcare staff on coping strategies. It is important to address this topic within ICUs, considering the intensified self-isolation to which patients are exposed, and understanding the impact that feelings of anxiety and loneliness can have on critically ill patients. This can contribute to more efficient action and support for individuals in coping strategies.

### Study limitations

The limitations of this study relate to issues such as geographical limitations and the number of participants. It is suggested that further studies be conducted in other states and countries so that we can truly understand whether cultural aspects, for instance, might alter the perspectives of this study.

### Contributions to nursing

This work provides valuable insights into the field of nursing, particularly regarding coping education. Nursing support was essential in the development of coping strategies by patients. Therefore, training the staff to practice humanization and implement specialized listening are important steps towards healthcare that also focuses on individuals’ mental health. Based on this study, it is recommended to develop evidence-based protocols, such as listening groups and psycho-spiritual support. Furthermore, these findings should guide public policies and clinical guidelines for post-COVID rehabilitation, promoting comprehensive and humanized care.

## FINAL CONSIDERATIONS

This study provided insights from people who were infected with COVID-19, required critical care, and survived. It highlights the predominance of post-COVID-19 sequels, such as fatigue, shortness of breath on exertion, difficulties with short-term memory, and symptoms of anxiety and depression. Furthermore, cardiac problems such as hypertension, lack of physical conditioning, and lack of energy were also evident.

Regarding problem-focused coping, weaknesses were observed associated with hospital infrastructure and overcrowding in units treating COVID-19. The fear of being transferred to a smaller hospital triggered the most stress among participants. The importance that patients placed on nursing professionals was evident; these professionals were essential in participants’ coping process, characterizing emotion-focused coping.

Considering the expressions of a greater appreciation for life after the traumatic situation, it was possible to infer that the experiences lived by the individuals were important in determining coping strategies. This was demonstrated by the desire to change aspects of life, such as opting for a healthier diet, more quality time with family, and valuing simple moments in life. Therefore, it is clear that it is necessary to invest in professional education regarding the coping process, with the aim of improving the quality of care provided in the emotional sphere within ICUs and extending to other healthcare units. Furthermore, programs that follow up post-COVID-19 are essential for better support in these patients’ rehabilitation.

## Data Availability

The research data are available within the article.
