# Supplementary material for: Coping with COVID-19 Intensive Care Unit admission
Source: Rev Bras Enferm. 2026 Jul 24;79:e20250382. doi: 10.1590/0034-7167-2025-0382 (PMC13403636; doi:10.1590/0034-7167-2025-0382)
Supplement: Translation

# Translation: Enfrentamento perante a internação em Unidade de Terapia Intensiva COVID-19

**DOI:** 10.1590/0034-7167-2025-0382pt

**Authors:** Mariane Carolina de Almeida, Maria Itayra Padilha, Maria Lígia dos Reis Bellaguarda, Stéfany Nayara Petry Dal Vesco, Jaime Alonso Caravaca-Morera

**Affiliations:** I Universidade Federal de Santa Catarina. Florianópolis, Santa Catarina, Brasil; II Universidade da Costa Rica. San José Province, San Pedro, Costa Rica

**Keywords:** Habilidades de Enfrentamento, COVID-19, Sequelas Pós-COVID-19 Aguda, Enfermagem, Unidades de Terapia Intensiva.

## Abstract

**Objetivos:**

analisar, à luz da Teoria de *Coping* de Folkman e Lazarus, as reflexões sobre a vida expressas nos relatos de experiência de pacientes internados na Unidade de Terapia Intensiva por COVID-19.

**Métodos:**

pesquisa qualitativa, por meio da História Oral Temática. Entrevistaram-se dez pacientes que internaram na Unidade de Terapia Intensiva COVID-19 de hospital universitário do sul do Brasil entre 2020 e 2021. Análise baseou-se no modelo teórico de Minayo.

**Resultados:**

participaram seis homens e quatro mulheres com idade média de 52 anos. Surgiram três categorias: “Sequelas pós-COVID-19”; “Qualidade da assistência e infraestrutura”; e “Valorização da vida e mudança de perspectiva”.

**Considerações Finais:**

durante a internação, destacou-se a importância dos profissionais de saúde, em especial os profissionais de enfermagem. Identificou-se uma mudança de perspectiva futura, com valorização da proximidade da família e maior apreciação da vida. Após a doença, foram identificadas dificuldades relacionadas à memória, ansiedade, fadiga e disfunções cognitivas.

## INTRODUÇÃO

Durante a pandemia de COVID-19 (coronavírus), doença causa pelo vírus SARS-CoV-2^(1,2)^, a população mundial sofreu com o medo de contrair a doença e de morrer, com as consequências do autoisolamento, com o impacto econômico, *etc*. Enfim, o desafio foi grandioso. Além dos sinais e sintomas muitas vezes inespecíficos^(3)^, os indivíduos que contraíram COVID-19 começaram a apresentar sequelas físicas e emocionais, conhecidas como síndrome pós-COVID-19^(4)^. Entre as sequelas mais citadas pela literatura mundial, destacam-se a anosmia persistente, dispneia em atividades de vida diária, falta de memória de curto prazo e problemas relacionados à saúde mental, como depressão, ansiedade e síndrome de estresse pós-traumático^(4,5)^.

Diante desses desafios, a enfermagem esteve presente em todos os níveis de atenção à saúde, prestando cuidados aos pacientes em todo o curso da doença^(6)^. Presenciaram-se enfermeiros atuando na linha de frente em Unidades Básicas de Saúde, Centros de Triagem para COVID-19, Unidades de Pronto Atendimento, hospitais de campanha, demais hospitais, entre outros ambientes de saúde^(7-9)^. Nos hospitais, a enfermagem precisou se adaptar ao novo setor de COVID-19, que, em muitas vezes, prestava atendimento em clínica médica e Unidade de Terapia Intensiva (UTI)^(10,11)^.

Os enfermeiros atuaram em condições de trabalho inóspitas nas UTIs, enfrentando sobrecarga e aumento da jornada de trabalho, falta de insumos e de recursos humanos, além do medo de contrair a doença e de serem estigmatizados caso fossem contaminados^(12)^. Ainda, com todas essas dificuldades, foi possível trabalhar com humanização e manter a qualidade da assistência. Por mais que o estresse relacionado à pandemia, por muitas vezes, tenha ocupado a mente das pessoas, nota-se que a sociedade aprendeu muito nesse período. Essa razão se deve ao reconhecimento do trabalho dos profissionais de saúde, ao aumento do número de atos solidários e ao enaltecimento da importância da família. Enfim, parece que se deu mais valor à vida^(4,13-16)^.

Nesse contexto, pensando em todo esse processo de estresse, (in)certeza, resiliência e aprendizado pós-COVID-19, está a Teoria de *Coping* (enfrentamento) de Folkman e Lazarus^(17)^, que nos remete às estratégias que são demandadas pelo indivíduo diante de situações estressantes. Em todo o processo pandêmico da COVID-19, houve estresse relacionado ao autoisolamento, medo da contaminação, medo da morte, medo de internar no hospital e medo de precisar de cuidados intensivos. Houve diversos elementos estressores durante a pandemia. Diante dessas situações, os indivíduos criam estratégias de enfrentamento. Algumas dessas estratégias podem ter foco na emoção, e outras, foco no problema^(18)^.

Embora as consequências físicas e emocionais da COVID-19 nas pessoas e o papel profissional da enfermagem durante a pandemia tenham sido amplamente documentados, ainda são escassos os estudos que exploram, sob a ótica da Teoria de *Coping* de Folkman e Lazarus, como os pacientes internados em UTIs significaram suas experiências e desenvolveram estratégias de enfrentamento diante da gravidade da doença e de suas sequelas. Isso instiga questionar: como os pacientes internados em UTIs por COVID-19 em hospital universitário do sul do Brasil expressam, à luz da Teoria de *Coping* de Folkman e Lazarus, suas capacidades de enfrentamento e estratégias de enfrentamento?

## OBJETIVOS

Analisar, à luz da Teoria de *Coping* de Folkman e Lazarus, as reflexões sobre a vida expressas nos relatos de experiência de pacientes internados na UTI por COVID-19.

## MÉTODOS

### Aspectos éticos

O estudo foi conduzido conforme diretrizes éticas nacionais e internacionais, sendo aprovado pelo Comitê de Ética em Pesquisa da Universidade Federal de Santa Catarina, cujo parecer está anexado à presente submissão. Foi desenvolvido de acordo com a Resolução n° 466/2012 do Conselho Nacional de Saúde. Todos os participantes assinaram o Termo de Consentimento Livre e Esclarecido.

### Tipo de estudo

Este estudo adotou uma abordagem qualitativa, com delineamento narrativo-sócio-histórico, utilizando fontes orais por meio da História Oral Temática^(19)^. O processo metodológico foi aprimorado seguindo o *checklist Consolidated criteria for REporting Qualitative research* ^(20)^, garantindo maior rigor e especificidade.

### Cenário do estudo

Foi desenvolvido em hospital universitário de município do estado de Santa Catarina (SC), aberto há 78 anos. É considerado um hospital estratégico do Ministério da Saúde, sendo um ponto de atenção à Rede de Urgências e Emergências. Desempenha um papel de destaque na comunidade macrorregional de saúde de Joaçaba, SC, sendo referência nas especialidades de oncologia e neurocirurgia para mais de 55 municípios, que somam mais de 600.000 habitantes. Apresenta capacidade de atendimento para aproximadamente 200 leitos. Conta com uma UTI geral, de 20 leitos, e uma UTI COVID-19, com dez leitos. Essa última UTI foi aberta em março de 2020, devido às necessidades dos pacientes com COVID-19, sendo suas atividades encerradas em maio de 2022. A UTI COVID-19 contava com dez leitos adultos, separados por cortinas divisórias de leito. O posto de enfermagem ficava ao centro da unidade. Cada leito possuía cama hospitalar, um monitor paramétrico, um ventilador mecânico, bombas de infusão, uma mesa de suporte, uma rede de oxigênio e ar comprimido.

### Participantes do estudo

Colaboraram com o estudo dez indivíduos que passaram por internação na UTI COVID-19 do Hospital Universitário Santa Terezinha, na cidade de Joaçaba, SC, entre 2020 e 2021. Foram incluídos na pesquisa pacientes que testaram positivo para COVID-19 e estiveram internados na UTI COVID-19 deste hospital, com idade superior a 18 anos e residência na cidade de Herval d’Oeste, SC. Foram excluídos da pesquisa pacientes internados na UTI COVID-19 deste hospital que residiam em outras cidades e com comprometimentos cognitivos que impossibilitassem a participação na entrevista. Foram convidados a participar do estudo pacientes que residiam na cidade de Herval d’Oeste, SC, o qual faz parte macrorregional de saúde de Joaçaba. O acesso à notificação dos casos foi feito através do setor de vigilância epidemiológica do município de Herval d’Oeste, SC. Os participantes foram selecionados intencionalmente.

### Coleta e organização dos dados

A coleta de dados se deu por meio de roteiro de entrevista semiestruturada, aplicada aos participantes no formato híbrido. A coleta de dados foi aplicada pela pesquisadora principal. Foram oferecidos dois formatos de participação - *online* ou presencial - em suas residências ou local de trabalho. Cinco participantes preferiram responder à entrevista de forma *online*, e cinco, de forma presencial. As questões tratavam das experiências de pessoas internadas com COVID-19, especialmente na UTI. Abordaram vida pessoal, profissional e de saúde antes da doença, além de sentimentos e vivências durante a internação, readaptação após a alta e impactos atuais, incluindo sequelas, mudanças na vida e apoio familiar no enfrentamento da doença. Todas a entrevistas foram gravadas pelo aplicativo *Zoom* ^®^ com a prévia autorização dos participantes. As entrevistas ocorreram entre junho de 2023 e junho de 2024. Após a transcrição dos dados no *Microsoft Word* ^®^, os arquivos foram enviados aos participantes via *WhatsApp* ^®^ para que eles pudessem fazer a validação da entrevista.

### Análise dos dados

Em seguida, prosseguiu-se à análise de dados, proposta por Minayo^(21)^, dividida em duas etapas: organização e fase exploratória; e interpretação e análise temática. Realizou-se uma leitura cuidadosa e exaustiva dos dados para obter uma visão geral e estabelecer relações iniciais, garantindo coerência interna. Foram registradas percepções preliminares, sendo iniciada uma análise crítica dos dados e mantendo a consistência das informações. Durante a exploração dos dados, as codificações foram realizadas, permitindo a formação de unidades de registro. Os códigos mais frequentes e com maior semelhança de significado contribuíram para categorização. Essa etapa incluiu o agrupamento das unidades de registro em subconjuntos, baseando-se em sua similaridade e correlação. A análise foi alinhada com a Teoria de *Coping.* A partir da análise, emergiram três categorias principais: “Sequelas pós-COVID-19”; “Qualidade da assistência e infraestrutura”; e “Valorização da vida e mudança de perspectiva sobre o futuro”. Para preservar o anonimato dos participantes, foram utilizados códigos alfanuméricos (P1, P2, P3, *etc*.), atribuídos na ordem cronológica das entrevistas.

## RESULTADOS

O estudo contou com a participação de dez indivíduos, dos quais seis eram do sexo masculino, e quatro, do sexo feminino. A idade dos participantes variou entre 34 e 71 anos, com uma média de 52 anos. No que diz respeito à raça/cor, seis se identificaram como brancos, e quatro, como pardos. Quanto ao estado civil, sete eram casados, dois, solteiros, e um era viúvo quando contraiu COVID-19. A religião predominante foi a católica, com nove representantes, seguida pela protestante. A média de permanência na UTI foi de 11,6 dias, com um tempo mínimo de internação de quatro dias e máximo de 33 dias.

### Sequelas pós-COVID-19

Os relatos sobre as dificuldades físicas e psicológicas enfrentadas após a infecção por COVID-19 destacam os desafios que os participantes vivenciam mesmo após a recuperação inicial. Além das sequelas físicas, muitos participantes da pesquisa relataram ansiedade, depressão e transtorno de estresse pós-traumático devido às experiências vividas durante a infecção e internação.

*Por exemplo, ontem mesmo pedi o nome de uma pessoa, e dez minutos depois, eu já tinha esquecido, então o esquecimento* [...] *é complicado eu lembrar de muita coisa, mas foi um momento bem difícil.* [...] *o folego, se eu exagerar, fica complicado descer ou subir escada rápido. Eu já sinto a diferença. É claro que não está 100%, né? Então, eu não posso fazer um exercício de me exigir muito, né. Hoje, eu faço caminhada, bicicleta, mas se eu for jogar uma bola, fica uma respiração; canso em dois toques.* (P2)

*Hoje, eu tenho uma dificuldade, porque leio dez páginas, e sai e volta, eu não consigo mais fechar se era capítulo, se era conto; eu não consigo mais acompanhar. E essa é uma das dificuldades que eu sinto das séries. Perdi um pouco dessa memória mesmo. Não tenho mais facilidade em gravar, e a leitura, várias vezes eu tive que recomeçar ou reler o capítulo. Perdi um pouco assim as coisas antigas.* (P3)

*Ficou, primeiramente, como posso te falar? Tu perde a resistência física; tu quer caminhar, e qualquer esforço já te faz, tem dificuldade para respirar, pelo menos, eu, esquecimento. Apareceu problema no coração, a pressão, a pressão nunca, mesmo tomando medicamento, volta e meia tem que ir na UPA porque não baixa.* [...] *me sinto com bastante ansiedade, olha do que estava no hospital está a mesma coisa.* (P5)

*Após a COVID-19, meu corpo nunca mais foi o mesmo. Fico sempre cansada, e esse cansaço parece que nunca vai embora. Mesmo para realizar as tarefas mais simples, como levantar da cama ou fazer o almoço, eu me sinto exausta. Minha cabeça também não está funcionando como antes; às vezes, ela dá umas travadas, e tem dias em que me sinto completamente perdida. Esqueço de coisas importantes e demoro mais para realizar tarefas que antes eram fáceis para mim. É como se a minha mente estivesse constantemente lenta e pesada.* (P7)

*O tempo com a COVID me deixou muito cansada, sem ânimo para nada. Sentia um cansaço extremo e uma memória que parecia não funcionar como antes. Precisei de fisioterapia para recuperar minha força, e fiz acompanhamento psicológico tanto dentro do hospital como em casa. Foi um processo lento e, muitas vezes, cansativo, mas eu me sentia grata por cada pequeno progresso que fazia. O cansaço dificulta as tarefas simples do dia a dia, e a sensação de que minha memória já não é mais a mesma.* [...] *me senti também muito ansiosa e triste durante o processo de recuperação, e precisei de acompanhamento com a psicóloga. Os pesadelos e a sensação de medo de que algo ruim pudesse acontecer novamente foram frequentes no começo, mas a terapia e o apoio da minha família têm me ajudado muito a superar isso.* (P8)

Os relatos mostram que, mesmo após a recuperação da COVID-19, os participantes enfrentam desafios físicos e psicológicos que comprometem sua qualidade de vida. Sintomas como cansaço, dificuldades respiratórias, perda de memória e lentidão cognitiva são comuns, assim como ansiedade, medo e estresse pós-traumático. Esses impactos afetam o dia a dia e a saúde mental, demandando acompanhamento contínuo e estratégias de enfrentamento, como terapias e apoio familiar.

### Qualidade da assistência e infraestrutura

As percepções dos pacientes sobre os serviços de enfermagem e infraestrutura hospitalar durante a pandemia de COVID-19 oferecem reflexões importantes sobre a qualidade do atendimento e as condições enfrentadas no período pandêmico. No momento de internação, os profissionais de enfermagem utilizaram palavras de conforto, encorajamento e esperança aos pacientes, além do cuidado humanizado, expresso pelos participantes em momentos de troca de roupas, alimentação, cuidados com sinais vitais. Ainda, os participantes do estudo relataram atitudes de presença constante, toques de cuidado, olhares atentos e posturas de escuta que reforçavam a humanização no atendimento.

*Teve um dia que eu acordei assim* [...] *umas três e meia da manhã ensopado de suor; as enfermeiras me trocando, porque eu estava todo molhado, pela saturação que baixou.* [...] *mas o atendimento deles foi muito bom, a alimentação, o cuidado, não dá para negar. Intubado eu não fui, mas eu fiquei com aquela máscara, que vinha no nariz e na boca, sabe. Mesmo assim, ninguém merece, não vinha ar.* [...] *a equipe da saúde não fazia nada para melhorar essa distância, só a única coisa é que eles ligavam para alguém da família para dizer como que estava. Eles pediam: “Quer que avise quem para nós dar o recado?”. Eles iam passando tipo, como a pessoa estava ou não. A única forma.* [...] *poderiam ter tratado melhor as pessoas. Alguns, eu sempre falo “alguns” porque não dá para generalizar, tem médicos ali que a gente lembra até hoje que foram pessoas boas, e outros, inclusive, melhor nem comentar, mas o atendimento em si foi de excelência.* (P2)

*Se alimentava bem, até era uma motivação quando entrava alguém com uma bandeja. Já dava assim. Aliás, assim, o tratamento da enfermeira, até escrevi um texto uma vez para uma enfermeira, que tinha colaborado com a gente; ela era secretária de saúde, enfermeira é quem que cuida, quem coloca você na cama e pergunta se está bem.* [...] *a médica é fundamental, mas quem lida com você, quem pede para trocar é enfermeira. Enfermagem é pouco valorizada financeiramente, professor da rede primária, e enfermeira, acho que ganha pouco. O sistema médico fazia parte do tratamento, mas a psicóloga ou uma assistente social fez a ligação de vídeo. Os profissionais ajudavam bastante conversando, história de vida, de onde você veio, isso eu acho que ajuda bastante. Talvez essa outra via que é pouco difundida na enfermagem. Recebi muito apoio; os médicos, assim, são um pouco mais técnicos, mas o pessoal da enfermagem e fisioterapia eram mais humanos.* (P3)

*Eu me lembro que uma enfermeira pediu se conseguia tomar banho, aí falei que conseguia, daí entrei pra tomar banho, e não, não consegui, começou faltar ar, comecei passar mal, aí a enfermeira teve que ir lá ajudar acabar o banho. Eu estava com o cateter só no oxigênio, aí já foi trocado e colocado aquela máscara com a bolsinha embaixo, e dali eu fui pra ala, pra ala do COVID* [...]. *Chegou o momento que ficava só uma enfermeira dentro da UTI cuidando o monitoramento lá dos aparelhos, e depois que vinha o resto ali para ajudar, dar uma olhada, porque lá fora, eu até me lembro de uma frase que a enfermeira me falou: “Parece um campo de guerra lá fora”.* [...] *em termos de equipe de saúde, olha... nota dez.* [...] *tinha um auxílio de psicóloga lá dentro também conversando com a gente. Foi bom, conversava, pediu como eu estava, e tocava no assunto da família; ajudava a distrair um pouco aquele tempo ali dentro, sempre dizia para pensar coisas boas, foi muito legal. Na verdade, dentro da área da saúde, ali nesse tempo que fiquei, foi, foi, assim, um atendimento de primeira, o empenho das pessoas, de todos* [...] *desde os médicos, enfermeiros, os técnicos, o psicólogo, a fisioterapeuta, o pessoal que atendia, que trazia o lanche, pessoal da limpeza, todos.* (P5)

*Os profissionais fizeram de tudo para gente se sentir acolhida, e isso fez toda a diferença. Eles foram maravilhosos, sempre vinham com uma palavra de carinho. Tinha uma enfermeira que sempre dizia: “Vai dar tudo certo”. Isso fazia a gente sentir que não estava sozinha. Eles sabiam que a gente precisava de apoio, e deram o melhor deles. Daí teve também a psicóloga que fez a chamada de vídeo; foi muito bom; senti minha família mais próxima.* (P6)

*Lá dentro, eles foram como uma família para mim: as enfermeiras, os médicos. Sempre tinham diziam que tudo ia dar certo. Eles sabiam como era difícil se sentir sozinho naquela situação, então faziam o possível para nos acolher e dar esperança. Mesmo nos momentos mais tristes, eles tentavam mostrar que ainda havia uma chance. O cuidado e a atenção deles foram muito importantes para que eu pudesse passar por isso tudo.* [...] *olha, na UTI, não lembro direito, mas depois, quando eu voltei na clínica, eles sempre faziam o que dava para que eu ficasse bem. São uns anjos mesmo que estavam lá para cuidar da gente. Eles foram verdadeiros anjos, sempre muito atenciosos e dedicados. Mesmo com tanta gente doente, eles faziam o que podiam para me confortar.* (P7)

*Foi um grande suporte durante meu tempo de internação e esse período de distância da família. Muitos deles tentavam me incentivar dizendo que eu estava melhorando, que estava progredindo, e isso me dava esperança. Algumas enfermeiras conversavam comigo sobre coisas do dia a dia, parecendo que eu nem estava no hospital. Sentia que Deus estava presente através desses profissionais que se dedicavam tanto a cuidar de mim e de tantos outros. A relação com os profissionais de saúde foi extremamente importante e trazia um conforto no meio daquilo tudo. Por mais que eu estivesse inconsciente durante uma boa parte do tempo que passei na UTI, quando comecei a acordar, percebi o cuidado e a dedicação de cada enfermeiro, cada médico. Eles estavam sempre presentes; era um cuidado a todo o momento.* (P8)

Quando se trata da infraestrutura hospitalar, os pacientes relataram suas impressões sobre o ambiente em que receberam tratamento durante a pandemia. As descrições incluíram tanto aspectos positivos quanto negativos, evidenciando a importância de um espaço adequado e bem equipado para o atendimento de saúde.

*Aí começou a bateria de exame. Naquele quarto, era a terceira cama, não tinha estrutura nenhuma, sabe? Aquela cama no depósito, assim, nem mobilidade a cama não tinha assim. Se dissesse que estava com dor na perna, eles colocavam travesseiro embaixo, ah, cobertor enrolado. Era assim meio que adaptado, recebia as visitas assim dos médicos, até que, no outro dia, passei a noite toda do dia 3, no 4 à noite, fui transferido para Chapecó.* [...] *era a terceira cama de um quarto; ela era adaptada do hospital; tinha outras pessoas que chegaram depois; tinha quatro ventiladores só, daí usei oxigênio, era cateter. O local, não tinha o que fazer, era um depósito, mas não pejorativo, não tinha lugar para o ventilador, não tinha lugar para oxigênio; tinha uns que faziam manual para dar uma forçada. Sinceramente, como eu já tinha trabalhado na saúde pública, eu digo para você: eu sinto que houve um certo abandono por não ter uns programas pós-COVID; não teve um programa oficial de pós-COVID; tive problemas hormonais, neurais e circulatórios; tive que fazer uma bateria de exames. Felizmente, eu tenho plano de saúde; comecei a procurar especialistas, neurologista, endo, nefro, monte; tive bastante complicação. Eu não tinha esses problemas antes, então foi pós-COVID.* (P3)

*Então, quando fui para o hospital, fiquei nessa sala branca até que me arrumassem um leito. Falaram que talvez eu fosse ter que ir para Campos Novos, mas eu não queria ir longe, mas, por sorte, “que tristeza” sorte minha, diz que um homem morreu, daí eu ia ter vaga, então fiquei ali, acho que na clínica por uns dois dias, e depois não vi mais nada.* (P7)

*Daí eles queriam me levar para Capinzal, daí eu disse: “Não, me deixem aqui”, daí fizeram um raio-X, e queriam me levar para Campos Novos, porque ali o hospital não tinha vaga de UTI. Eu disse: “Me deixem aqui”, já estava brigando com os homens lá. Daí depois que saiu o raio-X, falaram: “Não, vamos te internar aqui”. Eles diziam que eu estava mal, me deixaram lá embaixo, na maca, com oxigênio; eles queriam me transferir, daí não sei se era segunda ou terça-feira, eles queriam usar aquela máscara que empurra o oxigênio para eu conseguir respirar melhor, daí eles queriam me levar para Balneário Camboriú com a UTI móvel. Daí não sei, minha cunhada de Campos Novos conhecia a médica. Tentaram vaga na UTI ali, não deu certo, falamos com o doutor, que foi um anjo na minha vida também, que viu que uma pessoa faleceu, daí consegui ficar ali.* (P10)

Os relatos evidenciam o reconhecimento do cuidado da equipe de enfermagem e os desafios estruturais enfrentados. Há valorização do acolhimento, da escuta e do apoio emocional recebidos. Também se evidenciam limitações ligadas à superlotação, improviso de espaços e dificuldades no acesso a leitos de UTI, refletindo os impactos da crise sanitária na qualidade da assistência.

### Valorização da vida e mudança de perspectiva sobre o futuro

Muitos participantes relataram mudanças na forma de enxergar a vida após a recuperação da COVID-19, e ofereceram uma visão profunda sobre o impacto transformador que a doença pode ter nos indivíduos. Esses relatos destacaram a valorização de aspectos da vida que antes eram pouco valorizados, como as relações interpessoais, a saúde e a importância de momentos simples do cotidiano.

*Olha, do lado psicológico, uma coisa que eu já falava antes e falo agora: tem que aproveitar a vida hoje porque você não sabe do dia de amanhã. Coma e beba porque é isso que você vai levar. Aproveite, porque, depois que eu vi gente intubada do meu lado, morrendo no meu quarto, e o que eu levo de experiência hoje é que tem que aproveitar a vida. Amanhã você não sabe se vai levantar, ou se vai dar um infarto, ou um carro vai te pegar, entendeu? Então, eu sempre digo: “Tem que aproveitar”, porque é que nem eu falo: “Se você tem saúde, você fica bem”. Então, esse é ensinamento que eu digo: que tem que aproveitar.* (P2)

*Valorizar a vida e, claro, que aproveitar mais ela, mas a gente está numa função aqui que tenho certeza que, tenho mais seis, sete meses de mandato, e todo dia eu penso assim: “Quero, meu maior desafio é, é, estar motivado todo dia pra terminar a gestão do jeito que está trabalhando, fazendo melhor”.*[...] *é notável para mim isso, sabe, de realmente valorizar a vida, porque eu estive, na minha concepção, eu estava no outro lado já, mas tive uma segunda chance e algumas coisas que a gente poderia estar fazendo, fazendo errado.* (P4)

*A gente fica pensando, dá mais valor, valor, assim, paras coisas tipo alimentação. Você está lá, às vezes, estava lá no hospital, queria comer, não podia porque estava naquela situação. Tudo, tudo muda: sua forma de pensar das coisas. Tem coisa que não vale a pena perder tempo, não, muita coisa muda a opinião da pessoa, dar mais valor à vida. Uma coisa que eu prestava muito atenção é tipo assim: o simples fato de poder. No meu caso ali, estava lá na UTI, queria depois que voltei estava melhorzinho, queria ir ao banheiro, não podia, e você vê o quão fraco e frágil é o ser humano. E outra coisa, uma pessoa que tem independência faz tudo, e, do nada, assim, precisar de outras pessoas te ajudem para tudo, tudo mesmo, pra tomar banho, pra tudo, pra necessidades fisiológicas, tudo muda, é um aprendizado muito. A experiencia é ruim, mais o aprendizado é bastante de valor. Olha, a única coisa que, assim, as pessoas valorizar as pessoas que estão do teu lado, valorizar a família cada vez mais, aproveitar, aproveitar o tempo para ser feliz, não perder tempo com coisas mesquinhas, coisas que não te levam a lugar nenhum.* (P5)

*Gratidão de estar viva, de ter o meu marido e minha família comigo. Fiz muita fisioterapia; fui me recuperando devagar. Os bichinhos sempre perto, as meninas ligando todo dia* [...] *foi isso que me manteve firme. A gente aprende a valorizar cada momento. Mudou muita coisa. Acho que, depois de passar por isso, a gente vê a vida de outro jeito. Aprendi a valorizar mais as pequenas coisas: a saúde, a família* [...] *agora eu cuido mais de mim, ouço mais o meu corpo. A vida é tão frágil; a gente não pode perder tempo com coisa pequena.* (P6)

*Acho que, depois de passar por tudo isso, a gente vê a vida de um jeito bem diferente. Eu aprendi a valorizar muito mais a minha saúde e a minha família. Antes, eu acho que não dava tanta importância para essas coisas, mas agora é diferente. A gente tem que valorizar o que realmente importa: as coisas que fazem a diferença e que trazem alegria e satisfação.* (P7)

*Tento estar mais próxima da minha família, valorizando cada momento ao lado deles, e tento aproveitar a vida com mais gratidão e presença. Muitas coisas mudaram na minha vida. Primeiro, percebi a importância de cuidar melhor da minha saúde. Estou mais consciente sobre a necessidade de uma alimentação saudável, da prática de exercícios físicos. Também passei a valorizar mais os momentos simples ao lado da minha família e amigos. A experiência na UTI me fez refletir sobre a fragilidade da vida e a importância de viver o presente, com gratidão e propósito. A doença me ensinou a importância de nunca perder a esperança, de valorizar as pequenas vitórias do dia a dia, e de sempre manter a fé em Deus, mesmo nos momentos mais tristes.* (P8)

*A minha vida está melhor do que antes. A COVID e aquela fase que eu fiquei internado faz a gente pensar em muitas coisas, na vida, nas coisas simples que eu podia fazer, mas estava lá invalido, faz pensar muito na família, nossa, está tudo bem melhor.* (P10)

Os relatos indicam que a vivência da UTI COVID-19 despertou reflexões profundas sobre a fragilidade da vida, levando os participantes a ressignificar prioridades. Tornou-se essencial valorizar a saúde, a família e os momentos simples. A experiência de depender de cuidados intensivos e enfrentar o risco de morte reforçou sentimentos de gratidão, motivando mudanças na forma de viver, com mais presença, afeto e propósito.

## DISCUSSÃO

Com base nos achados, foi possível identificar sequelas persistentes nos pacientes após a COVID-19. Algumas delas são físicas, como dispneia, fadiga e problemas cardiovasculares; outras são cognitivas, como falta de memória de curto prazo; e outras são emocionais, como ansiedade e depressão. A compreensão dessas experiências permitiu analisar as reflexões sobre a vida desses pacientes que estiveram internados na UTI COVID-19 sob a ótica da Teoria de *Coping* de Folkman e Lazarus.

As sequelas da COVID-19 estão presentes em vários estudos, sendo as mais frequentes fadiga, falta de ar, palpitação, insônia, dor no peito, fraqueza muscular, perda de cabelo, dor nas articulações, dor abdominal, náusea ou vômito, mialgia, diminuição do apetite, dor de cabeça, distúrbio do olfato, distúrbio do paladar e tontura^(14-22)^. Em geral, essas sequelas são heterogêneas, ou seja, afetam vários sistemas biológicos, embora a falta de ar seja um dos sintomas mais citados nas literaturas^(23,24)^. Autores evidenciaram as sequelas pós-COVID em pacientes que passaram por cuidado crítico^(24)^, que giraram em torno de função física, cognitiva e psicossocial, como fraqueza, sintomas respiratórios prolongados, mudanças cognitivas, transtorno de estresse pós-traumático, ansiedade e depressão. Além disso, as sequelas pós-COVID têm se configurado como um desafio complexo, afetando múltiplos sistemas do organismo, e resultando em complicações respiratórias, cardiovasculares, neurológicas, psicológicas e musculoesqueléticas^(25)^. Como observado no presente estudo, os pacientes referem muitos dos sintomas pós-COVID que são descritos pela literatura, apenas não tendo sido observado estresse pós-traumático diagnosticado ou referido pelos participantes.

Em pesquisa que estudou enfermeiros que foram contaminados com COVID-19^(26)^, observou-se o relato de ansiedade durante o período de recuperação da COVID-19, dado esse que corrobora o presente estudo, uma vez que os participantes referem necessidade de acompanhamento psicológico e utilização de fármacos para tratamento em saúde mental. Esses dados destacam a importância de programas de acompanhamento dos pacientes pós-COVID, com objetivo de suporte à reabilitação dessas sequelas, trazendo melhor qualidade de vida a esses indivíduos.

O estresse vivenciado pelos participantes do estudo denota a estimulação pela busca de estratégias de enfrentamento. O medo, a ansiedade e a insegurança causaram estresse nos participantes, levando ao desenvolvimento de mecanismos inconscientes para lidar com a doença. Esses mecanismos são considerados *coping* emocional^(18)^, em que se buscaram apoio na família e religioso para enfrentamento desse processo.

Estudos^(27,28)^ indicaram que os profissionais de saúde também usaram o apoio e a comunicação com a família, amigos e colegas como seus principais mecanismos de enfrentamento. Mecanismos religiosos de enfrentamento, como a oração, foram relatados como um importante mecanismo de enfrentamento. Enfatiza-se, no presente estudo, o quanto a enfermagem foi exaltada no que se refere à competência técnica e de apoio psicológico. Os participantes parecem valorizar o serviço de enfermagem, enfatizando a importância desses profissionais e corroborando os dados de estudo^(29)^ em que os pacientes reconheceram a competência do profissional enfermeiro para a sua recuperação da COVID-19.

A valorização direcionada à equipe de enfermagem demonstra a importância da categoria profissional no processo de enfrentamento. Por meio do vínculo criado com o paciente, os profissionais passam confiança, tornando-se um ponto de segurança para esses pacientes, e isso, de forma inconsciente, ajuda no processo de enfrentamento da doença e da internação em UTI^(30)^. O foco está na regulação das emoções de acordo com o estresse vivenciado e os relacionamentos estabelecidos entre profissionais e familiares dos pacientes, bem como entre os próprios pacientes, por meio do apoio mútuo, da escuta ativa e da empatia. Além disso, as bases religiosas e espirituais forneceram suporte para o enfrentamento, que, em contraste com o *coping* focado na emoção descrito por Folkman e Lazarus^(18)^, traz regulação ao estado reflexivo contra o estresso^(31)^. Deve-se considerar que os profissionais também estavam em processo de *coping* durante a pandemia, talvez por estressores diferentes dos pacientes, mas que complementavam as situações vividas pelos usuários^(32)^.

Alguns participantes do estudo também referiram problemas relacionados à infraestrutura hospitalar que, em geral, foi precária. Esses problemas prescindem de enfrentamento, pois o sofrimento também está associado às más condições laborais^(30)^. O ambiente foi descrito como adaptado, como um “depósito”. Também ficou clara a ansiedade dos pacientes em relação à incerteza de conseguir um leito no hospital. O medo de ficar longe da sua cidade de origem e ter que ser encaminhado a um hospital mais longe ainda da família foi evidenciado pelos participantes. Os participantes de pesquisa^(29)^ referiram medo da superlotação hospitalar, da escassez de medicamentos e da falta de profissionais de saúde, de modo semelhante aos indivíduos da presente pesquisa.

Apesar das limitações apresentadas pelo ambiente hospitalar, a humanização e cuidado prestado pelos profissionais foram essenciais para o suporte físico e emocional dos pacientes. Pode-se aferir que os pacientes utilizaram o apoio dos profissionais como uma forma de *coping* emocional^(18)^. Utilizando palavras de apoio e incentivo, os indivíduos do estudo encontraram nos profissionais de saúde fonte de acalento no momento de estresse.

Foram observadas também mudanças de perspectivas sobre a vida, família e futuro. Alguns participantes referem um apreço maior sobre os momentos em família e valorização da liberdade em escolher o que se vai ingerir. Enfim, ficou claro que os pacientes valorizam mais os momentos simples da vida. Esses mecanismos podem estar envolvidos com a exposição a um estresse intenso, tendo em vista que estudos^(4-13)^ retratam mudanças de perspectivas após situações estressantes, como contaminação por COVID ou até mesmo internação em UTI.

Outro estudo^(29)^ descreve que os participantes expressaram que não devem permitir que o estresse da vida prejudique sua saúde, e ainda afirmaram que as questões financeiras, por exemplo, já não eram tão importantes para eles quanto antes da doença. A experiência hospitalar leva a uma “ressignificação” do corpo, com relatos semelhantes ao presente estudo de perda de resistência e mudanças na saúde física^(16)^.

Essa ressignificação da vida pode ser considerada uma estratégia de *coping* com foco no problema^(17)^, tendo em vista que, após uma situação estressante, os pacientes resolvem cuidar mais de sua saúde, reavaliando a qualidade de sua alimentação, a qualidade nas interações sociais e a importância da prática de atividade física. Essas são soluções simples para o problema enfrentado.

Esses achados prestam subsídios no sentido da oferta de educação às equipes de saúde sobre *coping*. É importante abordar esse tema dentro das UTIs, tendo em vista o autoisolamento intensificado ao qual o paciente fica exposto, entendendo o impacto que os sentimentos de ansiedade e solidão podem trazer aos pacientes críticos. Isso pode contribuir para a atuação de modo mais eficiente e como suporte ao individuo diante das estratégias de enfrentamento.

### Limitações do estudo

As limitações deste estudo se referem a questões como limitação geográfica e número de participantes. Sugere-se que outros estudos sejam realizados em outros estados e países, para que realmente possamos compreender se aspectos culturais, por exemplo, podem mudar perspectivas deste estudo.

### Contribuições para a área da enfermagem

Este trabalho presta subsídios para a área da enfermagem, principalmente no sentido de educação sobre *coping*. O apoio da enfermagem foi essencial no desenvolvimento de estratégias de *coping* pelos pacientes. Dessa forma, capacitar a equipe para que a humanização seja praticada e uma escuta especializada seja implementada são passos importantes para uma atenção à saúde que também foque na saúde mental dos indivíduos. Recomenda-se, a partir deste estudo, desenvolver protocolos baseados em evidências, como grupos de escuta e apoio psicoespiritual. Ainda, esses achados devem orientar políticas públicas e diretrizes clínicas para a reabilitação pós-COVID, promovendo cuidado integral e humanizado.

## CONSIDERAÇÕES FINAIS

Este estudo trouxe reflexões de pessoas que foram contaminados pela COVID-19, necessitaram de cuidado crítico e sobreviveram. Elucida-se a predominância das sequelas pós-COVID-19, como fadiga, dispneia ao esforço, dificuldades relacionadas a memória de curto prazo, sintomas de ansiedade e depressão. Ainda, ficaram evidenciados problemas cardíacos, como hipertensão, falta de condicionamento físico e falta de ânimo.

No que tange ao *coping* focado no problema, observaram-se fragilidades associadas à infraestrutura hospitalar e à superlotação das unidades que atendiam COVID-19. O medo de ser transferido para outro hospital, de porte menor, foi algo que desencadeou mais estresse nos participantes. Ficou evidenciada a importância que os pacientes demonstraram pela enfermagem, profissionais esses que foram essenciais no processo de enfrentamento dos participantes da pesquisa, caracterizando o *coping* focado na emoção.

Ao ser contemplada, nas expressões, uma maior valorização da vida após a situação traumática, permitiu inferir que as experiências vivenciadas pelos indivíduos foram importantes para determinar estratégias de enfrentamento. Isso foi demonstrado pela vontade de mudar aspectos da vida, como optar por uma alimentação mais saudável, mais tempo de qualidade com a família e valorizar momentos simples da vida. Sendo assim, fica claro que é necessário investir em educação profissional acerca do processo de enfrentamento, com o objetivo de melhorar a qualidade da assistência prestada no âmbito emocional dentro das UTI e se estendendo a outras unidades de assistência à saúde. Ainda, programas que acompanham o pós-COVID-19 são essenciais para melhor suporte na reabilitação desses pacientes.

## Data Availability

Os dados de pesquisa estão disponíveis no corpo do artigo.
